# Soil-Transmitted Helminths and Associated Factors among Pre-School Children in Butajira Town, South-Central Ethiopia: A Community-Based Cross-Sectional Study

**DOI:** 10.1371/journal.pone.0136342

**Published:** 2015-08-25

**Authors:** Teha Shumbej, Tariku Belay, Zeleke Mekonnen, Tamirat Tefera, Endalew Zemene

**Affiliations:** 1 Department of Diagnostic Laboratory, Butajira Hospital, Butajira, Ethiopia; 2 Department of Medical Laboratory Sciences and Pathology, College of Health Sciences, Jimma University, Jimma, Ethiopia; Universidade de Aveiro, PORTUGAL

## Abstract

**Background:**

Soil-transmitted helminths (STH) remain a major public health problem, particularly in tropical and sub-tropical regions of the world. Though infections are prevalent among all age groups, the world health organization (WHO) considers Pre-school age children (PSAC), school-aged children, and pregnant women as segments of population at high risk of STH morbidities.

**Objective:**

This study aimed at determining the prevalence and infection intensity of STH and associated factors among PSAC in Butajira Town, south-central Ethiopia.

**Methods:**

A community-based cross-sectional study was conducted from May to June, 2014 in Butajira Town. The PSAC were selected by systematic sampling technique and invited to participate in the present study. McMaster technique was employed for parasitological analysis of stool samples. Pearson’s Chi-square and Fisher’s exact tests were performed where appropriate to identify any association between STH infection and independent factors. Multivariate logistic regression model was fitted to identify independent predictors of STH among the PSAC. *P*-value less than 0.05 was considered statistically significant.

**Results:**

A total of 377 (with 96% compliance rate) PSAC were able to provide complete data (socio-demographic information and stool sample). The study showed that 23.3% (88/377) PSAC were infected with one or more species of STH. *Ascaris lumbricoides* was the most prevalent STH (14.9%) followed by *Trichuris trichiura* (6.4%). The overall infection intensity, expressed as geometric mean for *A*. *lumbricoides*, *T*. *trichiura*, and hookworms were 229, 178, and 154 eggs per gram of stool, respectively. The multivariate logistic regression model estimated that being in the age group of 36–47 months (AOR: 2.5, 95% CI: 1.2–5.3, *P* = 0.016), untrimmed finger nail (AOR: 3.2, 95% CI: 1.8–5.5, *P* < 0.001), and not washing hands before a meal (AOR: 3.0, 95% CI: 1.7–5.4, *P* < 0.001) were independent predictors of STH infections among the children.

**Conclusion:**

The present study showed that STH was a public health problem among PSAC in the study area necessitating annual deworming to control morbidities associated with STH. Besides, the existing health education program should also be strengthened to prevent re-infection.

## Introduction

Soil-transmitted helminths (STH) are among the most common chronic infections distributed throughout the world, disproportionately affecting poor population living in tropical and sub-tropical parts of the world [1, 2]. More than 1.5 billion people are estimated to be infected with STH [3], causing an estimated 4.98 million years lived with disability [4]. As a result, about 300 million people suffer from severe morbidity attributed to STH infections, resulting in 10,000–135,000 deaths annually [5].

Epidemiologic studies show that STH infections are aggregated with the majority of people harbouring few of the parasites while few people suffer from heavy intensity infection [6]. It is also known that morbidities associated with STH infection are directly related to the intensity of infection; hence people heavily infected with the STH experience the pathological sequels of infection. Preschool-aged children (PSAC), school-aged children, and women of child-bearing age are the segments of people at especial risk of STH morbidities [7, 8].

The consequences of STH infections include: malnutrition, iron deficiency anemia, impairment of physical and mental development, which ultimately retards educational advancement of the children and economic development of the nation [9, 10].

The geo-spatial distribution of STH is influenced by various factors like: poor environmental sanitation, lack of personal hygiene, and use of contaminated water [3] and other factors including age, socio-economic status, and occupation [11]. Ethiopia has one of the lowest quality drinking water supply and latrine coverage in the world [12], hence, intestinal parasites including the STH are widespread and cause significant morbidity in the country [13].

Currently, the primary control strategy of the STH mainly depends on mass drug administration (MDA) provided for population at risk of the diseases irrespective of their infection status; accompanied by health education and provision of clean water and sanitary facilities to sustain the effect of MDA [3, 14]. School-based deworming is the most commonly practiced strategy in developing countries. Even though deworming does not prevent re-infection, repeated deworming has been known to have a considerable impact on maintaining the intensity of STH infection to a minimal level, reducing the associated morbidities among infected individuals [15].

The frequency of MDA is based on the overall STH prevalence; the World Health Organization (WHO) recommends deworming once annually when the prevalence ranges between 20% and 50% or bi-annually when the prevalence is greater than 50% [15].

A population based study was conducted among mothers and infants of 1 year old in Butajira in 2006 and 2007 to determine the prevalence and risk factors for STH [16]. However, it cannot represent the age range for PSAC. Therefore, the present study was aimed to determining prevalence and intensity of STH and associated factors among PSAC in Butajira Town, south Ethiopia.

## Methods

### Study area and population

The study was conducted in Butajira Town between May and June 2014. The Town is located in Gurage Zone, Southern Nations, Nationalities, and People's Region of Ethiopia. It is located 135 km south of Addis Ababa, at approximately 7° 41' N latitude and 36° 50' E longitude. The town is located at an average altitude of 2,100 meters above sea level. The mean maximum and minimum annual temperature of the town is 30°C and 14°C, respectively. The annual rainfall ranges from 1138 mm to 1690 mm. According to Butajira Administration Family Health and Population Office, the total population of the town in 2013 was estimated to be 46,382, of which 11.7% (5425) were PSAC. At the time of this study, there were a total of 8,060 households (average household family sizes were 5.7) in the Town. Butajira Town has five *kebeles* (smallest administrative units in Ethiopia).

### Study design

**Fig 1:**
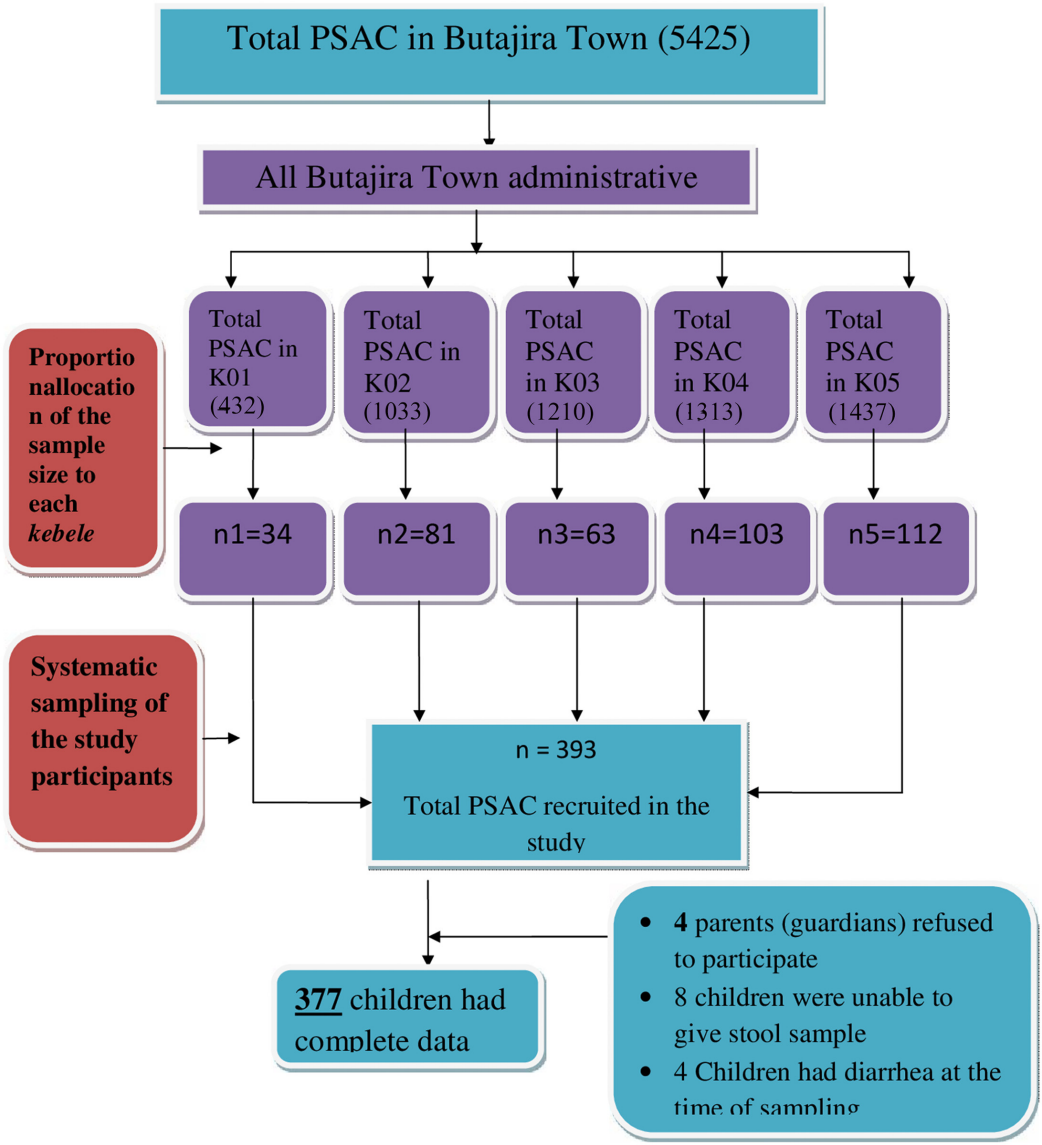
Flow chart of sampling, participation, and compliance of the Preschool aged children in Butajira Town, South-central Ethiopia.

A community-based cross-sectional study design was employed in the present study. A single population proportion formula was used to calculate the sample size required for the study; with an assumption of 5,425 total PSAC as a source population, estimated prevalence of 50%, 95% confidence level, 5% margin of error, and 10% non-response rate. Accordingly, the overall calculated sample size was 393 PSAC, which was then divided to each *kebele* proportional to the total number of PSAC in each *kebele*. Sampling frame was obtained by preliminary active house to house visit in collaboration with the health extension workers in each *kebele*. The study subjects were selected by systematic sampling technique as illustrated in Fig 1. The ratio of total number of PSAC found in particular *kebele* to sample size calculated in particular *kebele* was used as a sampling interval which was 12 in the present study. Hence, a single child per household was included in this study.

### Inclusion and exclusion criteria

Children aged between 12 and 59 months and whose parents or legal guardians signed the written consent were included in the study. Children who were not at home at the time of the survey visit and those who did not provide stool samples during first visit were revisited on the next day. Children who received anti-helminthic drug within one month prior to data collection and children having diarrhea at the time of stool collection were excluded from the study.

### Data collection

An interviewer administered semi-structured questionnaire was used to collect socio-demographic data of the children and factors associated with STH infection. The data were collected by urban health extension workers who can speak both *Amharic* and the local language (*Guraghaegna*).

### Sample collection and laboratory analyses

A fresh stool sample was collected from each study participants using pre-labeled clean, dry, and wide mouthed stool cups after which the samples were transported to Butajira General Hospital Laboratory. The approximate time lapse between sample collection and laboratory analysis was 2 hours. Parasitological examination of stool samples was done by McMaster technique as described elsewhere [17] for which single slide was examined by a trained laboratory technologist. Infection intensities of the STH were recorded and graded as light, moderate or heavy based on the number of eggs per gram (EPG) of stool according to WHO threshold [1].

### Data analysis

Data were entered into, cleaned, and analyzed by using SPSS for windows version 16 (Chicago, USA). The prevalence of STH was calculated as the ratio of number of children found positive for any STH species to the total number of children who provided complete data. Pearson’s Chi-square and Fisher’s exact tests were performed where appropriate to identify any association between STH infection and independent factors. Bivariate analysis was used to identify the associations of STH infections with independent variables. All variables with *P*-value <0.25 in the bivariate analysis were analyzed using multivariable logistic regression model. *P*-value less than 0.05 was considered statistically significant.

### Ethical considerations

The study protocol was reviewed and approved by Jimma University ethical review board. Permission was obtained from Butajira Town Administration Health Office and each *kebele’s* administration. Moreover, informed written consent was obtained from parents (guardians) of the children. Data collected from each child and results of laboratory tests were kept confidential. Children with positive stools for any STH infections were treated with single dose 100 mg Mebendazole tablets, according to Ethiopian medicines formulary recommendations [18] in collaboration with Butajira Administration Health Office.

## Results

### Socio-demographic characteristics

A total of 377 (96%) PSAC were involved in the present study. Age of the children ranged from 12 to 59 months with median age of 36 months (Inter-quartile range: 24–44). The age-group distribution showed that majority (31.8%) were in the age group of 24–35 months. More than half (56.2%) of the children were females and 40.6% of the households had family size of less than five. About half of the children’s mothers (guardians) had primary education while 26% had no formal education (Table 1).

**Table 1:** Soil-transmitted helminth infections in relation to socio-demographic characteristics of the pre-school aged children, Butajira, Southern Ethiopia, 2014.

Variables		Infection status		X^2^-test	P-value
		No. Positive (%)	No. examined (%)		
Sex	Male	41 (24.8)	165 (43.8)	0.372	0.542
	Female	47 (22.2)	212 (56.2)		
Age group in months	12–23	15 (14)	107 (28.4)	11.379	0.01**
	24–35	26 (21.7)	120 (31.8)		
	36–47	36 (33)	109 (28.9)		
	48–59	11 (26.8)	41 (10.9)		
Family Size	Below 5	53 (21.8)	224 (40.6)	0.896	0.344
	5 or Higher	35 (26.1)	153 (59.4)		
Mother’s (guardian’s) Education	No formal education	37 (37.8)	98 (26)	16.496	0.001**
	Primary	37 (20.2)	183 (48.5)		
	Secondary	7 (14.3)	49 (13)		
	Above secondary	7 (14.1)	47 (12.5)		
Mother’s (guardian’s) Occupation	Merchant	45 (23.8)	189 (50.1)	NA	0.54*
	Employed	9 (16.1)	56 (14.9)		
	House wife	30 (25.6)	117 (31)		
	Others ^b^	4 (26.7)	15 (4)		

^***b***^ student, farmer, daily laborer,

*Fisher’s exact,

**chi-square test.

### Prevalence and intensity of Soil-transmitted helminths

Overall, at least one species of intestinal helminths was detected in 104 (27.6%) of the children, of which 23.3% (88/377) had one or more species of STH (Table 1). Prevalence of STH infection was significantly highest among children in the age group of 36–47 months (33%), and whose mothers (guardians) had no formal education (37.8%). There was no significant difference of STH infection with regard to gender (*P* = 0.542), family income (*P* = 0.344), and family size (*P* = 0.389), and mothers’ occupation (*P* = 0.54) (Table 1).

The most frequently encountered intestinal helminth was *Ascaris lumbricoides* (14.9%) followed by *Trichuris trichiura* (6.4%) and hookworms (3.2%).. Majority (90.4%) of the infected children had single infection; while 9.6% had double infections. The overall infection intensity of STHs expressed as geometric mean among the study participants for *A*. *lumbricoides*, *T*. *trichiura* and hookworms was 229, 178, and 154 EPG, respectively. Most of the children had light infections and no heavy infection intensity was recorded among the children (Table 2).

**Table 2:** Infection intensity of Soil-transmitted helminths among Pre-school aged children in Butajira, south-central Ethiopia, 2014.

Infections intensity	Soil transmitted helminths		
	*Ascaris lumbricoides* No. (%)	*Trichuris trichiura* No. (%)	Hookworms No. (%)
Light	55 (98)	23 (95.8)	11 (91.6)
Moderate	1 (2)	1 (4.2)	1 (8.4)
Geometric mean (EPG)	229	178	154
Total	56	24	12

### Factors associated with soil-transmitted helminth infections

Children within the age group of 36–47 months (COR: 3.0, 95% CI: 1.5–5.9) and whose mother or guardian had no formal education (COR: 3.5, 95% CI: 1.5–8.5) had significantly higher prevalence of the STHs (Table 3). Prevalence of the STH infections was also significantly higher in children infrequently wearing shoes (COR: 2.2, 95% CI: 1.3–3.6) and those frequently playing in the soil (COR: 6.7, 95% CI: 1.9–22.8). Similarly, significantly higher prevalence of STHs infection was observed in children with untrimmed fingernails (COR: 4.4, 95% CI: 2.7–7.3) compared to those with trimmed fingernails (Table 3).

**Table 3:** Factors associated with soil-transmitted helminths infection among the preschool aged children, Butajira, south-central Ethiopia, 2014.

Variables		No. Positive (%)	No. Examined (%)	COR (95% CI)	P-value	AOR (95% CI)	P-value
Sex	Male	41 (24.8)	165 (43.8)	1.2 (0.7–1.8)	0.542		
	Female	47 (22.2)	212 (56.2)	1		-	-
Age in months	12–23	15 (14)	107 (28.4)	1		1	
	24–35	26 (21.7)	120 (31.8)	1.7 (0.8–3.4)	0.137	1.3 (0.6–2.9)	0.454
	36–47	36 (33)	109 (28.9)	3.0 (1.5–5.9)	0.001**	2.5 (1.2–5.3)	0.016**
	48–59	11 (26.8)	41 (10.9)	2.2 (0.9–5.4)	0.071	2.4 (0.9–6.6)	0.78
Family Size	Below 5	53 (21.8)	224 (40.6)	1			
	5 and above	35 (26.1)	153 (59.4)	1.3 (0.8–2.1)	0.34	-	-
Mother’s (guardian’s) Education	No formal education	37 (37.8)	98 (26)	3.5 (1.4–8.5)	0.007**	1.9 (0.7–5.7)	0.21
	Primary school	37 (20.2)	183 (48.5)	1.4 (0.6–3.5)	0.41	1.0 (0.4–2.7)	0.999
	Secondary school	7 (14.3)	49 (13)	0.9 (0.3–2.9)	0.933	1.0 (0.3–3.7)	0.982
	Tertiary education	7 (14.1)	47 (12.5)	1		1	
Mother’s (guardian’s) Occupation	Merchant	45 (23.8)	189 (50.1)	1			
	Employed	9 (16.1)	56 (14.9)	0.6 (0.3–1.3)	0.223	-	-
	House wife	30 (25.6)	117 (31)	1.1 (0.6–1.9)	0.717		
	Others*	4 (26.7)	15 (4)	1.2 (0.4–3.8)	0.803		
Shoe Wearing habit	Never	7 (29.2)	24 (6.4)	2.1 (0.8–5.1)	0.137	1.3 (0.4–4.5)	0.724
	Sometimes	50 (30.1)	166 (44)	2.2 (1.3–3.6)	0.003**	1.2 (0.6–2.3)	0.566
	Regularly	31 (16.7)	187 (49.6)	1		1	
Hands washing after defecation	Never	18(37.5)	48 (12.7)	3.2 (1.6–6.5)	0.001**	0.8 (0.3–2.0)	0.637
	Sometimes	38 (30.9)	123 (32.6)	2.4 (1.4–4.2)	0.001**	1.2 (0.6–2.3)	0.599
	Regularly	32 (15.5)	206 (54.7)	1		1	
Hands washing before meal	Sometimes	67 (36)	186 (49.3)	4.4 (2.6–7.6)	0.000**	3.0 (1.7–5.4)	0.000**
	Regularly	21 (11)	191 (50.7)	1		1	
Habit of playing in the soil	Never	4 (8.1)	37 (9.8)	1		1	
	Sometimes	25 (14)	179 (47.5)	1.8 (0.5–6.4)	0.341	0.8 (0.2–2.9)	0.704
	Regularly	60 (37.3)	161 (42.7)	6.7 (1.9–22.9)	0.002**	2.3 (0.6–8.3)	0.213
Habit of nail biting	Never	75 (22.9)	327 (86.7)	1		1	
	Sometimes	10 (22.7)	44 (11.7)	0.9(0.5–2.1)	0.975	0.7 (0.3–1.6)	0.37
	Regularly	3 (50)	6 (1.6)	3.4(0.7–16.9)	0.143	2.3 (0.3–15.0)	0.399
finger nail status	Trimmed	31 (13.2)	235 (62.3)	1		1	
	Untrimmed	57 (40.1)	142 (37.7)	4.4 (2.7–7.3)	0.000**	3.2 (1.8–5.5)	0.000**

* Student, farmer, daily labourer

** significant association

COR: Crude odd ratio, AOR: adjusted odd ratio, CI: 95% confidence interval

Moreover, STH infection was significantly associated with lack of hand washing habit after defecation, with higher infection among children infrequently washed their hands after defecation (COR: 2.4, 95% CI: 1.4–4.2) and those who do not wash their hands after defecation (COR: 3.3, 95% CI: 1.6–6.5). In addition, irregular hand washing before meal increased children’s odds for STH infection (COR: 4.4, 95% CI: 2.6–7.6) as compared to those regularly washing hands before meal (Table 3).

The multivariable logistic regression model estimated that children within the age group of 36–47 months were 2.5 times (AOR: 2.5, 95% CI: 1.2–5.3) more likely to get infected with STH than those within the age group of 12–23 months. Besides, children who had no regular practice of washing their hands before a meal were 3 times (AOR: 3.0, 95% CI: 1.7–5.4) more likely to be infected with STH than those who had regular practice. The odds of STH infection was 3 times higher (AOR: 3.2, 95% CI: 1.8–5.5) for children who had not trimmed their finger nail as compared to those who did (Table 3).

## Discussion

In recent years, the control of neglected tropical diseases (NTDs) has got significant attention by many governments, donors, and international agencies [19]. In Ethiopia, MDA for the control of STH is in its early stage of implementation as the mapping of the STH and schistosomiasis was completed in 2013 [20].

The present study attempted to determine the prevalence, intensity, and factors associated with STH among PSAC in Butajira Town. A community-based survey provides pertinent information regarding the burden of STH in a community, and enables evidence-based decision to be made in due course of intervention [19].

The overall prevalence of STH in the present study was 23%. According to WHO, STH endemic areas classifications, there are three categories in line with application of MDA: i) high transmission (where prevalence is > 50%), ii) moderate transmission (where prevalence is between 20%- 50%), and iii) low transmission (where prevalence is < 20%) [2, 21]. Accordingly, the study area would be classified into the moderate transmission group calling for annual deworming.

The prevalence of STH in the present study corroborates the findings of a study from Southwest China [22]. However, it is lower than the findings of other studies [23–26].

The differences in prevalence and distribution of the STH among the different communities might be due to variation in both host-specific and environmental factors that may affect transmission of STH infections. These factors may include: population heterogeneity, age, genetics, polyparasitism, time of study, parasitological technique used, personal hygiene practices, climate, and altitude among others [27, 28].

In the present study, *A*. *lumbricoides* was the most common species of STH recovered from the children. This is consistent with the findings of similar studies conducted elsewhere [23, 29, 30]. On the contrary, other studies reported *T*. *trichiura* as the predominant STH among PSAC [31–33]. The variation might be due to differences in environmental factors such as climate, topography, surface temperature, altitude, soil type and rainfall which have a great impact on the distribution of STHs [27, 28].

Prevalence of STHs in the present study increased with age. This trend was also observed in other studies conducted elsewhere [23, 25, 34, 35]. This phenomenon might reflect age related change in exposure to STHs infection. Though most children do start walking by the age of 13 months, they might not be strong enough to go outdoor and get infected until the age of 2 years. This developmental factor might be the reason for the slightly lower prevalence in those who were 12–23 months of age; and an increasing trend thereafter.

In this study, poly-helminthic infection rate was observed in 2.6% of the study participants. This figure is comparable with the findings of other studies [36, 37]. However, it is very low compared to what was reported elsewhere [31, 35]. The difference could be due to variation in sample size, diagnostic methods, and the number of specimens investigated to rule-out diagnosis. The McMaster technique, employed in the present study, has been found to be effective in the detection and quantification of the STH but not preferred for detection of polyparasitic infections [25].

Various possible factors considered to affect STH infection were assessed in this study. Untrimmed finger nail and not washing hands before meal were the key factors significantly associated with STH infections in the present study; which is in agreement with other studies conducted in Ethiopia and also other parts of the world [31, 35, 38, 39]. This highlighted the need for integrated control of STH, i.e. deworming should be backed-up with health education to prevent re-infection. This is because; the only way of re-infection with STH is exposure to the infective stages from contaminated environment as these parasites do not multiply within the human host [15].

The present study had its own limitations; the study design being cross-sectional, could not underline the causal association between the STH and socio-economic and demographic characteristics. Nutritional status of the children was not assessed as to investigate its association with STH infections. We used the McMaster method, which has previously been shown to be user friendly, robust and accurate for enumeration of STH, but less sensitive when intensity of infection is low. Besides, it is not suitable for detection of *Strongyloides stercoralis*, which is one of the STH [25]. Moreover, since only single stool specimen was collected from each child there might be significant underestimation of prevalence of STH because of the temporal variation in egg excretion over hours and days. Finally, the time elapsed before stool examination, as the samples were collected from field and transported to the laboratory, might result in underestimation of hookworm spp. as a result of rapid desiccation of hookworm eggs in the stool samples [22]. Nevertheless, the fact that it was a community-based study and the systematic sampling of the PSAC from the community would make it possible to generalize the findings to the whole PSAC in the study area.

In conclusion, the present study showed that STH was a public health problem among PSAC in the study area necessitating annual deworming to control morbidities associated with STH. Poor personal hygiene such as not washing hands before meal and untrimmed fingernail were the key factors significantly associated with STH infections among the children. This highlighted the need for integrated control of STH in the study area. Therefore, the existing health education program should be strengthened to sustain the effect of deworming.

## Supporting Information

S1 Data Set Supporting data set for which mean, median and standard deviation is calculated. (XLSX)
